# Flow as a Key Predictor of Subjective Well-Being Among Chinese University Students: A Chain Mediating Model

**DOI:** 10.3389/fpsyg.2021.743906

**Published:** 2021-11-16

**Authors:** Jun Wu, Mei Xie, Yao Lai, Yanhui Mao, Laszlo Harmat

**Affiliations:** ^1^School of Foreign Languages, Southwest Jiaotong University, Chengdu, China; ^2^School of Economics and Management, Southwest Jiaotong University, Chengdu, China; ^3^Institute of Applied Psychology, Psychological Research and Counseling Center, Southwest Jiaotong University, Chengdu, China; ^4^Department of Psychology and Behavior Sciences, Zhejiang University, Hangzhou, China; ^5^Department of Psychology, Linnaeus University, Växjö, Sweden

**Keywords:** flow experience, academic self-efficacy, self-esteem, subjective well-being, Chinese university students

## Abstract

The present study investigated a conceptual model by testing flow experience and subjective well-being of university students during Coronavirus Diseas-19 (COVID-19) *via* considering their underlying mechanisms of academic self-efficacy and self-esteem. A total of 1,109 Chinese university students completed a questionnaire containing scales of subjective well-being, flow, academic self-efficacy, and self-esteem. Results yielded from the structural equation modeling analysis indicated a significant and positive association between flow experience and subjective well-being, and such an association was sequentially mediated by academic self-efficacy and self-esteem. Findings also provided empirical evidence for the proposed model highlighting the significant role of flow experience at the higher educational context in predicting subjective well-being of Chinese university students, and how such a relation can be supported by suggested mediating roles academic self-efficacy and self-esteem played.

## Introduction

Subjective well-being can be defined as an overall evaluation of one’s own life and affective feelings regarding emotional experience (Diener et al., 2017). Therefore, it has two components: cognitive component of life satisfaction or contentedness and affective component (positive vs. negative affect) of emotional experience, which make individuals feel productive and able to cope with life stressors to achieve well-being (Diener and Seligman, 2004; Delle Fave et al., 2011). Research has indicated that higher level of subjective well-being of university students is associated with better physical, mental, and academic growth (Delle Fave and Bassi, 2016). As a younger generation, well-being of university students exhibits great impacts on science and technology development for any given society within any country, since they play special social roles in carrying out important missions for the development of a nation compared with other general populations (Slutske, 2005).

A very recent challenge posed by the new coronavirus disease 2019 (COVID-19) that has threatened well-being of individual (Pagliaro et al., 2021; Peng et al., 2021), also threatened physical and mental health of university students, disordered their campus life by changing the lifestyle (Charles et al., 2021). Under such circumstances, university students have experienced unprecedented challenges (Brooks et al., 2020), such as anxiety (Cao et al., 2020; Chi et al., 2020; Huang and Zhao, 2020), distress (Hasan and Bao, 2020), and disengagement in learning activities (Khlaif et al., 2021), which in turn have threatened their well-being. Given these ongoing challenges faced by university students, it is paramount for the government, educators, and psychologists to act for maintaining or promoting the well-being of this population (Qiu et al., 2020; Tian et al., 2020; Wu and McGoogan, 2020).

Research on factors associated with the mental health status of students during COVID-19 has been well documented (Cui et al., 2021; Pagliaro et al., 2021). For instance, university students who were more satisfied with the educational environment would feel much happier (Anderson et al., 2019; Booker and Perlin, 2020; Bélanger and Ratelle, 2020). Numerous records have suggested that facilitating an enjoyable flow experience is a good strategy for promoting academic performance of university students (Shernoff, 2010; Csikszentmihalyi and Larson, 2014; Mao et al., 2020) and promoting their well-being, directly or indirectly (Cantor and Sanderson, 2003; Diener and Diener, 2009; Bassi et al., 2014; Coffey et al., 2016; Daw et al., 2016; Tse et al., 2021). Previous works also found that an increase in academic self-efficacy and self-esteem is associated with an increased optimal enjoyable experience of flow (Choi and Kim, 2013), which in turn contributes to subjective well-being (Diener and Diener, 2009; Delle Fave and Bassi, 2016; Wang and Fowler, 2019). However, we are not aware of any studies that have simultaneously assessed the joint contributing roles of these factors (i.e., flow, academic self-efficacy, and self-esteem) for subjective well-being of university students, especially in the context of COVID-19. To this end, we aimed to investigate a conceptual model by testing flow experience of university students and their subjective well-being relation *via* considering the underlying mechanisms of academic self-efficacy and self-esteem within such a relationship.

### Development of the Model

#### Flow and Subjective Well-Being

Though there is no unified definition of subjective well-being that can be adopted by all institutions (Travia et al., 2020), in consensus, psychological studies on subjective well-being are mainly focused on two perspectives: hedonic and eudaimonic. The hedonic perspective focuses on personal happiness and good mood, which includes life satisfaction and positive effects (Diener et al., 2009). While the eudaimonic perspective focuses on outcomes, such as the purpose of life, personal growth, and meaning in life (Waterman, 1993, 2007). For instance, recent work has found that eudaimonic orientation of university students moderates the happiness benefits of prosocial activities like money donation (Lai et al., 2020); living in truth to one’s true self gives rise to eudaimonia, which certainly leads to subjective well-being (SWB) (Waterman, 2011; Cui et al., 2021). People achieve and maintain hedonic well-being through the eudaimonic process (Lent, 2004), as people are likely to achieve a happy and satisfaction in life by engaging in meaningful activities and working through their personal goals (Mao et al., 2016). Such engagement, according to the father of positive psychology (Seligman, 2002), is actually the optimal experience called “flow.”

Flow describes the affective and cognitive state when people are immersed in the current activities at hand (i.e., academic study), they are completely involved, entirely concentrated, and fully focused with capacity in information processing at the speed of about 110 bytes per second (i.e., vs. 40 b/s when doing academic work while talking), having a sense of time distortion that either time passes quickly or stops (Csikszentmihalyi and Csikszentmihalyi, 1992; Nakamura and Csikszentmihalyi, 2002). For many years, the flow has become an increasingly important asset in a variety of fields (Mao et al., 2016). For example, the flow was found to significantly reduce anxiety of university students through increased academic self-efficacy and promoted self-esteem in higher education (Mao et al., 2020). Flow related to work has been proven to be able to promote positive organizational outcomes, such as work performance, organizational citizenship behavior, and subjective well-being (Bakker, 2008; Fullagar and Kelloway, 2009; Soriano et al., 2021). Interestingly, research has also demonstrated that flow experience is a highly unstable “optimal experience” in all walks of human activities (Csikszentmihalyi, 1975; Mao et al., 2016), as the individuals keep stretching their skills in coping with fluctuated challenges from the outside world on their way of personal growth. Flow is varied greatly at the within-person level, indicating that it changes at an individual’s different stages of life span (i.e., from pre-school to college years) and with its dynamic nature being stressed (Fullagar and Kelloway, 2009; Debus et al., 2014). In this regard, Ceja and Navarro (2009, 2011, 2012) have found that the flow state within human beings tends to follow a disordered pattern, that is, flow experience shows a constant fluctuation during each day and does not go steady over time. What experiencing flow means and what effects flow potentially for university students in the world have been verified by many researchers (Chang et al., 2017; Soulliard et al., 2019). Although the positive impact of flow on subjective well-being is well established, and flow is found to be a universal experience especially in work and study (Bonaiuto et al., 2016; Mao et al., 2016), the precise underlying mechanism of the flow effect on subjective well-being, however, remains unclear. The accumulating works on flow demonstrate that flow moderates the link between quarantine length and well-being in Chinese participants (Sweeny et al., 2020) and many research records on subjective well-being of college students in the time of COVID-19 (Cui et al., 2021; Genç and Arslan, 2021), we accordingly hypothesize that:

**H1**. *A disposition to experience flow is positively associated with the subjective well-being, the more flow experience, the more subjective well-being of the Chinese university students.*

#### The Potential Mediation Effects of Academic Self-Efficacy and Self-Esteem

Self-efficacy refers to the confidence of an individual to take actions toward his or her goals (Bandura, 1977). When university students do not have the confidence to pursue their desired academic goals, they may lose hope or find no meaning in life, because the study is one of the major daily activities they pursue. Self-efficacy encourages increasing personal skills and abilities *via* finding ways to manage stress and to control challenging situations on one’s own (Muris, 2002), therefore, it can enhance mental health and one’s well-being (Kim, 2003). When one’s skill competence meets the challenge of outside or external social environment, s/he will experience flow from the activity at hand (Mao et al., 2016). Within the context of higher education, academic self-efficacy is considered to be an important determinant of academic performance of university students and their personal development (Honicke and Broadbent, 2016; Talsma et al., 2018), because academic self-efficacy is positively related to optimal enjoyment of flow brought about by study/learning activities (Joo et al., 2015; Mesurado et al., 2016). For example, when students control their learning situation and completely immerse themselves in their own academic activities, they feel a strong sense of time distortion, and therefore flow tends to occur more frequently (Fullagar et al., 2013). With coping as a mediator between personality and stress outcomes (Knoll et al., 2005), self-efficacy activates the response to be assertive when facing a challenging situation, resulting in the encouragement of one’s well-being (Eskin, 2003). When university students feel that they have the confidence to deal with stressful and uncomfortable situations, they manage the situations and move on (Bandura, 1997). A study about academic adjustment and life satisfaction in Portuguese college students *via* a longitudinal design has found that academic self-efficacy and environmental support are predictive of goal progress and academic adjustment, of which academic adjustment is predictive for global life satisfaction of students, and academic self-efficacy and positive affect are found to be reciprocally related to one another (Lent et al., 2009). Academic self-efficacy is also an important factor in predicting academic resilience of Latino college students (Cavazos et al., 2010). Taken together, given that an individual with higher academic self-efficacy can better cope with difficult situations like trying to alleviate anxiety (Mao et al., 2020), it is much more likely that academic self-efficacy can be positively associated with subjective well-being.

Theoretically, the higher the level of one’s flow (or optimal experience), it is more likely that one can set a clear goal, the steadier control one has over his task, the stronger immersion and pleasure one can feel (Csikszentmihalyi, 1975, 1990). As an important part of the flow, one’s independence or freedom when engaging in activities is repeatedly found to be able to increase positive affect (Saavedra and Kwun, 2000) and motivation (Fried and Ferris, 1987), which in turn promotes academic self-efficacy. As academic self-efficacy is reflected widely in the daily life of university students especially during the challenging time of COVID-19 (Alemany-Arrebola et al., 2020), an individual who has more frequent flow experience may feel increased confidence and a loss of self-awareness during academic activities, in this sense, we predict that:

**H2.** *Academic self-efficacy mediates the positive relationship between flow and subjective well-being so that individuals who have experienced more flow will have stronger academic self-efficacy and thus more subjective well-being.*

Self-esteem refers to the extent to which individuals like, value, accept, and respect themselves at a general or global level (Rosenberg, 2015). It is defined as a positive attitude one holds toward him- or herself and is a self-assessment of one’s own value (Jordan et al., 2020). Self-esteem, as a sense of self-worth, is closely associated with subjective well-being and a number of other adaptive outcomes (Seligman and Csikszentmihalyi, 2000; Diener and Diener, 2009; Diener et al., 2015). Prior research has indicated that an individual with higher self-esteem enjoys more positive feelings, in comparison with those who hold lower self-esteem (Martín-Albo, 2007). Numerous research records have dated that one’s self-esteem can strongly predict positive mental health, such as life satisfaction and well-being (Mann et al., 2004; Lehtinen et al., 2006; Wang and Fowler, 2019). Besides, self-esteem is also important for maintaining objective physical health (Stinson et al., 2008). Self-esteem begins to shape in childhood and continues to change and develop throughout the lifespan (Rubin et al., 2002). Studies from a large national sample of young adolescents in the United States (aged between 12 and 16 years) demonstrate that lower level of self-esteem is associated with a number of modifiable risk factors, such as a lower level of team sports participation and poor school performance, and vice versa (McClure et al., 2010). In contrast, a higher level of self-esteem helps to cope with stressors by accumulating available coping resources that support mental health (Crabtree and Rutland, 2001; Taylor and Stanton, 2007).

As for the relationship between one’s self-esteem and his or her flow experience, contradictory findings coexist in research records. For instance, some have argued that self-esteem is the antecedent of flow: greater self-esteem encourages an increased level of flow when engaging in digital games (Choi and Kim, 2013). Whereas others have suggested that it is the lower level of self-esteem (instead of higher self-esteem) that allows people to have a higher level of flow experiences when surfing on the Internet, playing computer games, and immersing in mobile phones (Khang et al., 2013). Understanding such a paradox needs to consider the nature of game experiences based on flow theory (Csikszentmihalyi, 1975, 1990): if the activity itself is to be carried out with a meaningful goal toward personal growth, self-actualization and harmonious integration of the individual are achieved in the social context, then higher self-esteem brings about higher flow, which is consistent with the eudemonic value of well-being. If, however, playing digital games is to avoid negative feedback and unpleasant situations from others and the outside world, then, the activity itself is reckoned as an anti-social activity, thus engaging in such anti-social activity is to express low self-esteem, though one may still have a sense of optimal enjoyment with hedonic pleasure (Delle Fave, 2013). To date, the above concrete evidence supporting a link between self-esteem and flow is generated from the digital activities over the social media, however, whether such a paradox relationship applies to academic activities within a university context, needs further investigation, though the flow concept has been introduced in designing educational software/games.

The previous section has indicated that self-esteem is the antecedent of flow (Khang et al., 2013). However, it can be the other way around, because the flow has important potential in promoting personal development, such as developing one’s character strength (Wells, 1988), that being said, experiencing more frequent flow in everyday life brings about higher self-esteem (Asakawa, 2010). Recent findings have indicated that because of the impact of COVID-19 lockdown, youngsters who are exposed to more social network sites show lower self-esteem (Vall-Roqué et al., 2021). Flow experienced more and frequently during academic activities at university is associated with increased self-esteem (Mao et al., 2020), which, in turn, mediates the association between positive psychological functioning (i.e., optimism and social support) and subjective well-being (Kong et al., 2013; Duy and Yıldız, 2019). However, little is known about whether the challenge of COVID-19 that brought about in-campus lifestyle changes of university students, such as a sudden shift to online learning due to the pandemic quarantine (Anderson et al., 2020; Pagliaro et al., 2021), may pose an impact on their experience of both positive functioning of flow and self-esteem, which may in turn affect subjective well-being. We therefore assume that:

**H3.** *Self-esteem mediates the positive relationship between flow and subjective well-being.*

Previous sections have provided supports on the positive association of flow with academic self-efficacy and of flow with self-esteem. Prior empirical work has also supported a positive association between self-esteem and self-efficacy beliefs (e.g., D’Amico and Cardaci, 2003). However, there are inconsistent findings about the association between self-esteem and self-efficacy, yet no records can be traced in the literature to address their joint contribution to well-being. Specifically, there are inconsistent results about the association between self-esteem and self-efficacy. In fact, self-esteem concerns one’s judgment of his or her self-worth, whereas perceived academic self-efficacy is concerned with the judgment of personal competencies and capabilities in academic study. For example, one may judge himself inefficacious in a certain activity without investing his self-worth in that activity, thus, he will not suffer from any loss of self-esteem for being inefficacious (Bandura, 1997). Considering the academic activities in higher educational context at university, students may perceive themselves with increased self-esteem if they achieve academic goals by stretching capabilities with increased skills (Delle Fave and Bassi, 2016), and such positive self-esteem may in turn induce them to perceive their academic capabilities as more positive (D’Amico and Cardaci, 2003). However, although cognitive ability and conscientiousness have been found to predict performance, less is known about whether and when certain mediating variables help explain these relationships (Chen et al., 2001). There are no studies, to our best knowledge and insofar, on the roles of academic self-efficacy and self-esteem played in uncovering flow and subjective well-being relationship. Nevertheless, a recent work may provide an implicit clue that flow can predict academic self-efficacy, which in turn predicts self-esteem, and subsequently predicts reduced anxiety (Mao et al., 2020).

Other indirect evidence can also support such a sequential mediation. For instance, Stupnisky et al. (2013) proposed that if students can maintain both a higher sense of control and self-esteem at university, they will experience more positive well-being and will perform better academically afterward. Odaci (2013) found a positive (but not significant) correlation between the belief of postgraduate students in academic self-efficacy in research and their self-esteem. Data from the German Aging Survey revealed that programs aiming at increasing optimism, self-esteem, and self-efficacy might be helpful to maintain subjective well-being (Hajek and König, 2019). Taken together, all these evidences lead us to propose our assumption that:

**H4.** *Academic self-efficacy and self-esteem sequentially mediate the relationship between flow and subjective well-being.*

**FIGURE 1:**
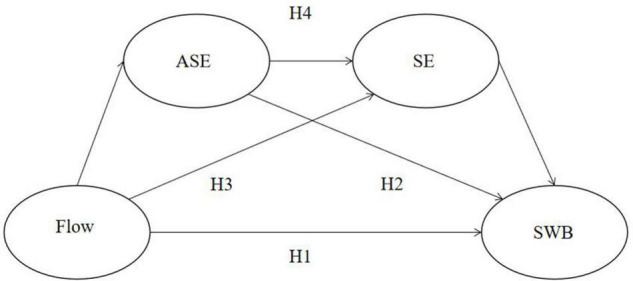
Proposed conceptual model. ASE, academic self-efficacy; SE, self-esteem; SWB, subjective well-being; H1, Flow-SWB; H2, Flow-ASE-SWB; H3, Flow-SE-SWB; H4, Flow-ASE-SE-SWB.

The proposed model is therefore indicated below in Figure 1.

## Materials and Methods

### Participants and Procedures

A large sample of university students in southwest China (*N* = 1109, *M*_*age*_ = 21.8, SD = 2.5) participated in the present survey, of which 237 were male and 872 were female (see Table 1 for details). All the participants were given the online informed content, were confirmed their voluntary participation, and were notified the confidentiality prior to answering all the questionnaire items. The invited participants were encouraged to share the survey link with their peers within and outside their university. The questionnaire took approximately 5–10 min to complete. Ethical review and approval were waived for this study by the Institutional Review Board of Southwest Jiaotong University according to the guidelines of the Declaration of Helsinki, as the study involving questionnaire survey did not involve personal privacy issues, yet issues on psychological or physical harms to participants. The data-gathering phase started from December 13 to December 28, 2020.

**TABLE 1:** Social demographic features of participants (*N* = 1109).

**Variables**		**Percentages**
Gender	Male	21.37%
	Female	78.63%
Age	15–25	96%
	26–35	4%
Discipline	Literature	40.67%
	Engineering	32.91%
	Science	11.09%
University year	First-year Graduate	24.53%
	Freshman	15.42%
	Senior	14.16%
Single child	Yes	50.05%
	No	49.95%
Marital status	Single	62.67%
	In love	35.17%
Parenthood status	Yes	0.45%
	No	99.55%
Family monthly income	Above 8,000	31.74%
	Below 4,000	26.78%
	4,000–6,000	22.36%
	60,000–8,000	19.12%
Personal income	Family support	74.75%
	Scholarship and subsidies	16.86%
	Part-time job	6.76%

### Measures

All measures were administered in the Chinese language, going through translation and back-translation procedures from the original English version. Answers to each item of our adopted measure were registered on a Likert-type scale ranging from 1 (It doesn’t describe me at all) to 7 (It describes me exactly).

#### Subjective Well-Being

Subjective well-being was assessed by the 5-item Satisfaction With Life Scale (SWLS; Diener et al., 1985), which has been widely used for testing the core component and the cognitive aspect of subjective well-being (e.g., Zanon et al., 2014; Jovanović and Brdar, 2018; Chaves et al., 2021; Kong et al., 2021). Since the first item is similar to the second item under Chinese context in our pilot test, while the factor loading for the fourth item was pretty low, these two items were excluded in further analysis. The sample item was “*The conditions of my academic life in this university are excellent*.” We averaged responses to create an overall individual subjective well-being index, in which a higher score on this measure indicated greater subjective well-being (Arrindell et al., 1999). Cronbach’s alpha coefficient for the present sample was 0.800.

#### Flow

The 7-item Swedish Flow Proneness Questionnaire (SFPQ; Ullén et al., 2012) was designed as a self-report measure of how frequently the participant has flow experiences pertained to each of the three typical divisions of activities (work, maintenance, and leisure time). SFPQ has been used in numerous studies (Mosing et al., 2012; De Manzano et al., 2013; Gyurkovics et al., 2016; Niksirat et al., 2019; Liu and Csikszentmihalyi, 2020). Three items were deleted because of the low factor loadings, therefore, yielded four items representing typical flow characteristics for a single dimension that was pertained to academic study in a university context for the frequency of flow. The sample item was “*I often feel that my skills/abilities completely match what I do no matter how difficult it is*.” Scores of each participant including reversed items were coded, higher scores represented higher frequency of flow experience. The reliability of the scale was good (Cronbach’s α = 0.813) in the present sample.

#### Academic Self-Efficacy

We adopted an 8-item Academic Self-Efficacy Scale (Stagg et al., 2018) that has been used previously by Mao et al. (2020) in a Chinese adolescent sample with a reported good internal consistency (Cronbach’s α = 0.837). However, in the present work, we deleted three items after the pilot test due to its lower factor loadings conducted from confirmatory factor analysis. Thus, the responses of each participant were registered based on five finalized items of academic self-efficacy scale, which covered the three principal facets: learning efficiency, examination, and learning processes (e.g., *I am confident in my ability to manage my time effectively in study*). The higher the score, the greater the level of the academic self-efficacy. For the present sample, the reliability was good (Cronbach’s α = 0.879).

#### Self-Esteem

Self-esteem was measured based on the well-recognized 10-item Rosenberg Self-Esteem Scale (RSES; Rosenberg, 1965) on which the participants indicated the extent to which they felt themselves to possess good qualities, to accept their own characteristics, and to have achieved personal success or experienced failure. This scale has been widely used (e.g., Mao et al., 2020). Because of the low confirmatory analysis (CFA) factor loadings yielded on three items, data were analyzed based on seven items. The sample item was “*I take a positive attitude toward myself*.” The greater the accumulated score, the greater the self-esteem. Cronbach’s alpha coefficient was 0.911.

### Analytic Strategy

Data were analyzed *via* SPSS 26.0 and AMOS 21.0. First, descriptive statistics, correlational indices among variables, and validity and reliability of constructs were conducted in SPSS. Recommended by researchers (Anderson and Gerbing, 1988), the subsequent two-step procedure was followed to test the proposed conceptual model in AMOS. Step 1, the measurement model was tested ensuring that the observed indicators could represent the four latent variables well. On the premise that the measurement model was fine, finally, step 2, a series of possible structure models were tested *via* structural equation modeling with the estimation of maximum likelihood (ML) method. Specifically, a set of fitting indices were considered for the model fit: root-mean-square error of approximation (RMSEA < 0.08), standardized root-mean-square-residual (SRMR < 0.08), comparative fit index (CFI > 0.90), normative fit index (NFI > 0.90), and goodness-of-fit index (*GFI* > 0.90). A non-parametric bootstrap method (5,000 samples) was used to test the significance of the mediating effects, with a 95% CI failing to contain zero, indicating a significant mediation effect (Hu and Bentler, 1999).

## Results

Table 2 presents the descriptive statistics of all study variables. As predicted, all study variables were positively and significantly correlated with each other (with *p* < 0.01). Specifically, the flow was positively associated with academic self-efficacy, self-esteem, and subjective well-being. It was worth noting that the results of the ANOVA taking gender or age group as the factor yielded no significant effect on our study variables. Therefore, social demographic variables (i.e., gender and age) were excluded from the subsequent analyses.

**TABLE 2:** Descriptive statistics and correlational indices among variables.

**Variable**	**Mean**	**SD**	**Flow**	**ASE**	**SE**	**SWB**
Flow	3.9853	0.98142	1			
ASE	4.5713	1.02560	0.568^**^	1		
SE	4.7391	1.01558	0.521^**^	0.652^**^	1	
SWB	3.9468	1.01190	0.475^**^	0.495^**^	0.484^**^	1

**p < 0.01. ASE, academic self-efficacy; SE, self-esteem; SWB, subjective well-being.

### Validity and Reliability of Constructs

**FIGURE 2:**
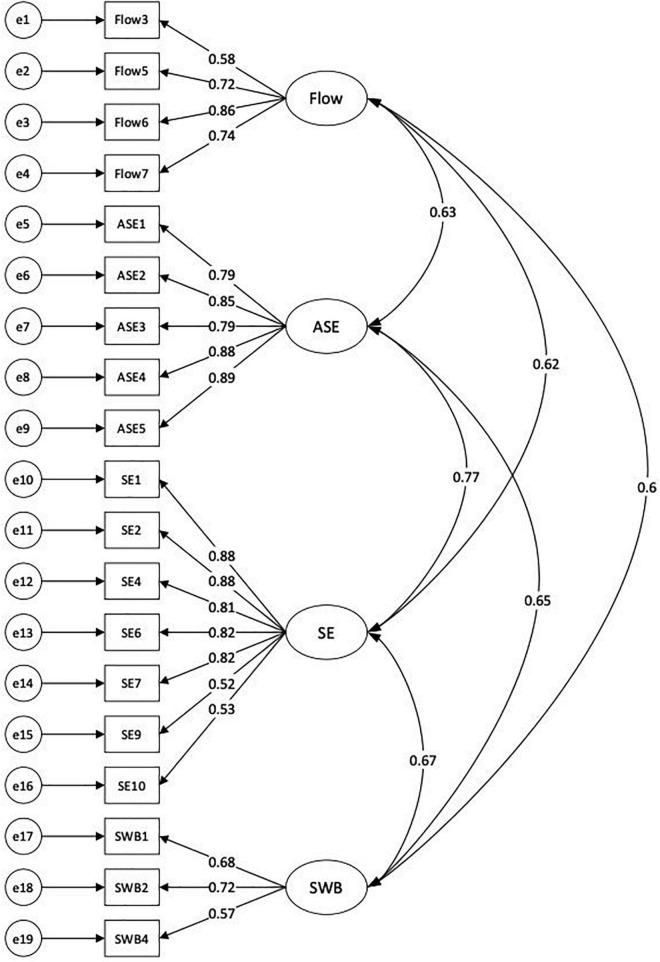
The measurement model. ASE, academic self-efficacy; SE, self-esteem; SWB, subjective well-being.

We carried out Kaiser–Mayer–Orkin (KMO) test and Bartley spherical test (*p*-value) in SPSS before conducting CFA. According to recommendations, KMO > 0.5 with Bartley spherical test (*p* < 0.05) would indicate that the questionnaire is of good structural validity (Kaiser and Rice, 1974). As indicated in Table 3, the KMO values for flow, academic self-efficacy, self-esteem, and well-being were all above 0.7 (*p* < 0.001), indicating that this measurement model had good structural validity and was suitable for CFA. The truncation value of the Cronbach’s *α* (internal consistency) higher than 0.8 represents the good reliability of the measurement model. As indicated in Table 3 and Figure 2, all of the CFA factor loadings of the observed indicators corresponding to respective latent variables are above 0.522, indicating that the extracted common factors are highly representative of the study variables.

**TABLE 3:** Test of construct validity and reliability.

**Construct**	**Item**	**CFA factor loading**	**KMO**	**Bartley spherical test**	**Cronbach’s α**
Flow	Flow3	0.582	0.780	0.000	0.813
	Flow5	0.721			
	Flow6	0.860			
	Flow7	0.744			
ASE	ASE1	0.788	0.854	0.000	0.879
	ASE2	0.847			
	ASE3	0.791			
	ASE4	0.879			
	ASE5	0.887			
SE	SE1	0.876	0.835	0.000	0.911
	SE2	0.877			
	SE4	0.807			
	SE6	0.816			
	SE7	0.820			
	SE9	0.522			
	SE10	0.532			
SWB	SWB1	0.681	0.670	0.000	0.800
	SWB2	0.718			
	SWB3	0.566			

*ASE, academic self-efficacy; SE, self-esteem; SWB, subjective well-being; CFA, confirmatory analysis; KMO, Kaiser–Mayer–Orkin.*

### The Measurement Model

The measurement model that composed of four latent variables with 19 observed indicators, as depicted in Figure 2, revealed a satisfactory fit to our data: *x*^2^ = 343.136, *df* = 121, *p* < 0.001; RMSEA = 0.041; RMR = 0.052; CFI = 0.985; GFI = 0.977; NFI = 0.969.

### Test of the Structural Model

Tests of hypotheses *via* structural equation modeling analysis were conducted using the ML estimation method to examine the hypothesized mediational model (driven by theory and literature), adjusted model (driven by data), and three possible alternative models (possible pathway relations that might exist though not driven by the theory and literature) to find an optimal model solution. As indicated in Table 4 and followed by Hu and Bentler (1999) for the cut-off indices for the goodness-of-fit (*x*^2^/*df* < 3, RMSEA < 0.08, CFI > 0.9, NFI > 0.9, IFI > 0.9, and GFI > 0.9), Model 4 fitted the data better in comparison to alternative models. To compare our proposed model with alternative models (see Table 4), we also relied on the Akaike information criterion (AIC), which states that models with lower AIC should be preferred to models with higher AIC, as it compares the parsimony of models based on the same covariance matrix. Taken together, results demonstrated that Model 4 adjusted from the conceptual model, which yielded a chain mediation, was the optimal fitting model for our data.

**TABLE 4:** Fitting indices for the proposed model and adjusted model (*N* = 1,109).

**Model Fit Indices**	**χ^2^**	***df***	**χ^2^/*df***	**GFI**	**NFI**	**IFI**	**RMSEA**	**AIC**	**AGFI**	**CFI**	***P***
Model 1: Flow-SE-ASE-SWB	2,499.917	148	16.891	0.835	0.832	0.841	0.120	2,583.917	0.788	0.840	0.000
Adjusted Model 1	482.752	137	3.524	0.955	0.968	0.977	0.048	588.752	0.938	0.977	0.000
Model 2: ASE-Flow-SE-SWB	2,702.965	148	18.263	0.820	0.819	0.827	0.125	2,786.965	0.769	0.827	0.000
Adjusted Model 2	451.960	123	3.674	0.958	0.970	0.978	0.049	585.960	0.935	0.978	0.000
Model 3: ASE -SE-Flow-SWB	2,499.917	148	16.891	0.835	0.832	0.841	0.120	2,583.917	0.788	0.840	0.000
Adjusted Model 3	473.449	128	3.699	0.957	0.968	0.977	0.049	597.449	0.936	0.977	0.000
Proposed conceptual model (Figure 1)	2,380.908	146	16.308	0.843	0.840	0.849	0.118	2,468.908	0.795	0.848	0.000
Model 4 (Figure 3)	392.121	131	2.9934	0.96	0.974	0.982	0.042	510.121	0.947	0.982	0.000

*χ^2^/*df* 3, RMSEA < 0.08, GFI > 0.9, AGFI > 0.9, NFI > 0.9, CFI > 0.9 indicating good fit. RMSEA, root-mean-square error of approximation; NFI, normative fit index; GFI, goodness-of-fit index.*

**FIGURE 3:**
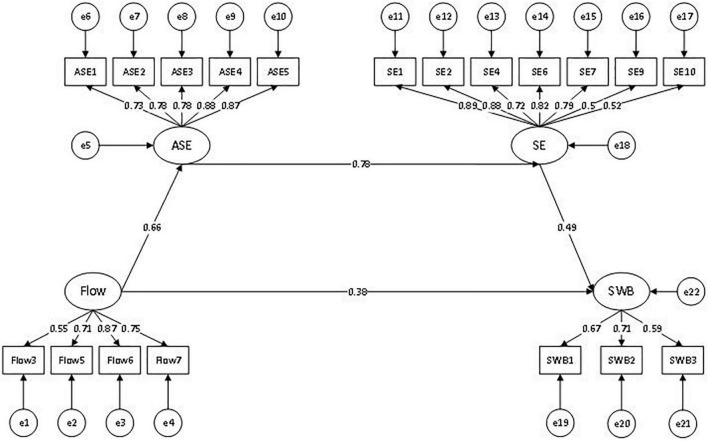
Adjusted structural equation model.

The complete structural equation model estimated by ML is shown in Figure 3. A chain mediation of academic self-efficacy and self-esteem between flow and subjective well-being was found. With a non-parametric (5,000 samples) bootstrap method used to test the significance of the mediating effects, it was demonstrated to have statistical significance (*p* = 0.01). As would be discussed subsequently, the general goodness-of-fit and other internal quality indices of the adjusted model (Model 4) were confirmed to be optimal. Therefore, we continued to perform tests of mediation and multigroup analyses based on the diagram, which are presented in Figure 3.

### Mediating Effects and Total Effects

Despite the fact that all paths of the model in Figure 3 were confirmed to be statistically significant, further tests of mediating effects and total effects were essential to identify the predicting effects that flow and other psychological constructs exerted on subjective well-being.

Results based on standardized estimates of indirect effects and direct effects by ML are shown in Table 5. It was clear that all indirect and direct effects were significant, with *p*-values less than 0.01. Table 5 reports two mediating-effect tests and two direct-effect tests. In one respect, self-esteem played a mediating role in the relationship between flow and subjective well-being, while academic self-efficacy mediated the link between flow and self-esteem. Self-esteem mediated the relation between self-efficacy and subjective well-being. It can be concluded that self-esteem played a fully mediating role in two related associations, while academic self-efficacy partially mediated the link between flow and self-esteem. These results confirmed our hypotheses H1 and H4. From Table 5, it was also noted that of all the predicting effects for subjective well-being, self-esteem had the strongest total effect, followed by flow (optimal experience), while academic self-efficacy was the weakest predictor for subjective well-being. Accordingly, the diagram of the adjusted structural model is displayed in Figure 3.

**TABLE 5:** Standardized indirect and direct effects and 95% CIs.

**Path**	**Standardized estimate**	***p***	**Bias-corrected percentile**		**Results**
			**Lower**	**Upper**	
H1: Flow→SWB	0.381	0.000	0.271	0.490	Supported
Flow→ASE	0.663	0.000	0.605	0.717	Supported
w→ASE→SE	0.519	0.000	0.463	0.574	Supported
H4: Flow→ASE→SE→SWB	0.253	0.000	0.204	0.309	Supported

*SWB, subjective well-being; ASE, academic self-efficacy; SE, self-efficiency.*

## Discussion

The present study introduced a conceptual framework to uncover the bounded relationships between flow, academic self-efficacy, self-esteem, and subjective well-being, under the challenge of the spread of COVID-19, which has posed severe threats to the well-being of individuals especially the youngsters of university students. Findings obtained from a large sample (*N* = 1109) of Chinese university students confirmed that flow experience was a peculiarly positive and complex state of conscious experience that can be achieved in academic activities in a higher educational context (Csikszentmihalyi and Larson, 2014; Mao et al., 2020). It is worth noting that though our sample was not balanced by gender or age, the primary ANOVA test *via* the factor of either gender or age did not yield any significant difference on study variables. Findings that flow was massively reported *via* academic activities irrespective of gender or age in present work were consistent with prior literature (Csikszentmihalyi, 1975, 1990; Bonaiuto et al., 2016; Mao et al., 2016; Mao et al., 2020; Peng et al., 2021). Besides, such findings were in line with previous results that were generated from other populations (Bakker, 2008; Fullagar and Kelloway, 2009; Delle Fave and Bassi, 2016; Peifer et al., 2020; Peng et al., 2021). Our result that flow had a significant positive impact on subjective well-being of university students is confirmed by our hypothesis H1, while was also consistent with prior studies in western culture (Cantor and Sanderson, 2003; Tamannaeifar and Motaghedifard, 2014; Ilies et al., 2017; Kim and Hall, 2019).

Findings confirmed that flow was positively associated with academic self-efficacy, greater flow experience, and academic self-efficacy being reported at university tended to be associated with greater learning progress (Hong et al., 2017) and greater well-being (Kim, 2003; Joo et al., 2015; Mesurado et al., 2016). But academic self-efficacy did not play the full mediating role between flow and well-being, when interpreting this result, it is important to consider variables described in other studies, such as behavioral markers connected to vulnerability to stress (Natovová and Chýlová, 2014), perceived social support (Wang and Castañeda-Sound, 2008), and so on, which may be key when it comes to explaining why there are other variables in the chain relation, i.e., between academic self-efficacy and subjective well-being. Other variables, such as personality factors and character strengths, may also have effects on such a relationship, as earlier studies found that neuroticism showed a negative relationship with flow proneness (e.g., Ullén et al., 2012; Ross and Keiser, 2014; Tse et al., 2021) and subjective well-being, self-esteem, and self-efficacy (Judge and Bono, 2001; Sobol-Kwapinska, 2016).

Findings on academic self-efficacy and self-esteem confirmed that the relationship between the two remained a matter of contention that the direction of these variables is likely to be bidirectional. Though other studies have pointed out the contribution of self-esteem to academic self-efficacy (e.g., Di Giunta et al., 2013). However, our data led us to the conclusion of the prediction effect of academic self-efficacy to self-esteem, and this is consistent with recent findings from the Chinese university student samples (Mao et al., 2020).

Results on hypothesis H4 confirmed that self-esteem was positively associated with subjective well-being (Diener and Diener, 2009; Kong et al., 2015), which resembled various studies that had reported university students in some populations having higher levels of subjective well-being in terms of more flow experiences and greater self-esteem (Asakawa, 2010), but the best fitting model from the current study did not support the full mediation effect of self-esteem between flow and subjective well-being, one clue that might help to explain this finding is that although low self-esteem is conceptually distinct from some factors, such as social anxiety, depression, or loneliness, evidence exists for moderate correlations among these variables (Leary and MacDonald, 2003). Hence, a particular note should be taken into consideration for interpreting such a result, as the relationship between flow and self-esteem and the possible mediators need further verification (Mao et al., 2020). As we anticipated, academic self-efficacy and self-esteem play a great sequential mediation role in the process of the impact of flow on the subjective well-being of university students, that is, the higher the academic self-efficacy and thus higher self-esteem of university students, the greater the impact of flow on their subjective well-being. Our research supported previous findings on the positive association among flow, academic self-efficacy, and subjective well-being (Eskin, 2003; Lent et al., 2009), flow, self-esteem, and subjective well-being (Tse et al., 2021); among flow, academic self-efficacy and self-esteem (Pellas, 2014; Kim and Park, 2018); and among academic self-efficacy, self-esteem and subjective well-being (Hajek and König, 2019), and further extended these findings to a Chinese university students’ sample. As consistent with previous studies (Odaci, 2013; Stupnisky et al., 2013; Hajek and König, 2019; Mao et al., 2020), the present investigation verified the chain mediation effect. It should also be noted that previous research results have already shown the correlation between enhancing subjective well-being by considering flow experiences, personal traits, and values along with confidence in learning ability and perceived academic control (Stupnisky et al., 2013; Bajaj et al., 2016). However, in contrast to our assumptions, the results revealed a uniquely full and sequential mediation effect among these four variables. We also wish to reassert the need to include both school variables (Weber et al., 2016) in this analysis and factors from the socio-family environment (Lyubomirsky et al., 2005), so as to depict a full picture of subjective well-being of university students.

### Implications

The present study has suggested that a higher level of flow is directly and positively associated with subjective well-being of university students, and flow is also significantly and positively predictive for subjective well-being through a chain (or sequential) mediation effect of academic self-efficacy and self-esteem. Therefore, implications of this work relate to strategies for facilitating flow, academic self-efficacy, and self-esteem for promoting well-being of university students. It is worth noting that though COVID-19 has posed threats and challenges to academic life of university students that has been greatly shifted into the online mode because of governmental implemented quarantine and self-isolation measures (Anderson et al., 2020; Pagliaro et al., 2021), exposure to the more online social network when performing learning activities did not lower down university students self-esteem (in contrast with prior findings, Vall-Roqué et al., 2021), academic self-efficacy or subjective well-being, as long as there was optimal enjoyable flow experience in learning activities.

First, flow theory acts as an option, which can be implemented in terms of everyday ordinary activities, with very simple and clear ways of knowing by which we can create simple and available strategies for improving human psychological sustainability of commonly and optimally experienced activities and contexts (such as academic study). Therefore, it represents a way to foster students resilience in the face of challenging, demanding, and stressing requests (Csikszentmihalyi and Csikszentmihalyi, 1992; Mao et al., 2020). Proposed strategies for flow can start with setting clear and achievable learning goals, especially in the period of COVID-19 that both self-isolation and quarantine require self-discipline without any supervision as students are usually in the university campus. Therefore, strategies, such as writing a to-do list, for every tomorrow before sleep at night can ensure a step-by-step feedback checking if goals are achieved or not, make each day fulfilled and leave anxiety or boredom behind (Peifer et al., 2020). Strategies are also needed to encourage university students to learn soft skills and new knowledge to foster interpersonal relations and stretch resilience in coping with potential challenges on the way to approach academic goals (Csikszentmihalyi, 1975). Supervisors, course instructors, and university administrates could provide online virtual teaching resources for students to stretch their skills and competencies for balancing academic challenges on the way beyond the COVID-19 pandemic.

Second, regarding the proposed strategies for academic self-efficacy and self-esteem, efficient teaching and psychological guidance should be provided to prepare students for the situation of long-term online distance education, improve adaptive capacity of students, reduce their cognitive anxiety, and enhance self-efficacy in academic activities (Mao et al., 2020). In this way, social network sites’ detrimental effects on students could be exacerbated during the pandemic (Vall-Roqué et al., 2021), due to the great impact of enjoyable flow being experienced, either alone or with other peers, or online that could bring students with identity at different levels (personal, social, or place) (Bonaiuto et al., 2016; Mao et al., 2016). The greater sense of self at various levels may encourage great self-academic efficacy. Therefore, university students should control the frequent and aimless use of digital tools (i.e., mobile, personal computer or other software), instead, make academic learning more meaningful and purposeful so as to transfer the hedonic happiness into eudaimonic well-being. University administrative staff, together with supervisors and course teachers, should collaborate together in building a harmony atmosphere through various online teaching platforms that can facilitate enjoyable academic learning to build strong self-confidence and courage in coping with academic demands and stress in their daily routine during COVID-19 (Cui et al., 2021).

In a word, a positive attitude toward one’s self and learning activity, a positive cognitive evaluation of self and academic situation, together with positive engagement—flow (Waterman, 1993), all help enrich internal resources of students to cultivate and increase frustration tolerance, promoting their resilience and preventing their burnout, therefore to make their academic in an upward spiral (Delle Fave and Bassi, 2016), and so that facilitate subjective well-being and personal growth of university students (Delle Fave et al., 2011).

### Limitations

There are several limits that warrant notice. First, the nature of cross-sectional data prevents us to make causal inferences, therefore, longitudinal studies, time series cross-lagged analyses (i.e., dairy study), or strict experimental designs are profitable in future work to specify the directionality of the relationships among the variables, such as the proposed mediational effect of academic self-efficacy and self-esteem. Second, the model tries to approximate the reality but cannot draw a whole picture with limited factors in consideration, thus future work may try to involve more indicators with more crucial exogenous variables to improve the structure model and to depict a better subjective well-being picture that is close to reality, or alternatively, apply qualitative comparative analysis (QCA) to depict a full picture with a comprehensive perspective. As our data were self-reported from measures regarding the last 3 months pertaining to a length of the COVID-19 period, we therefore used trait-based flow frequency measure (instead of state-based flow). On this basis, we only adopted the core dimension-cognitive dimension of the subjective well-being measure (Kong et al., 2013), excluding the affective dimension as the emotional affect of the students fluctuates across time during the days of COVID-19. Though a final yield of three items may not fully represent general well-being, the positive and significant role of dispositional flow in facilitating subjective well-being of university students has never been challenged. Additional research is likely needed to identify other mediators within this relationship, such as personality factors (i.e., big five personalities), which can be associated with flow proneness, self-esteem, and self-efficacy. As subjective well-being is an extremely sophisticated phenomenon involving a large number of factors that may cover economics and sociology besides education, a proposed model with more factors and with better methodological solutions may help gain deeper insights into mechanisms for facilitating flow experiences in predicting subjective well-being of university students.

## Data Availability Statement

The raw data supporting the conclusions of this article will be made available by the authors, without undue reservation.

## Ethics Statement

The studies involving human participants were reviewed and approved by Institute of Applied Psychology, Southwest Jiaotong University. The patients/participants provided their written informed consent to participate in this study.

## Author Contributions

YM and JW conceptualized the idea. YL and JW carried out data collection and analyses. MX and YM drafted the manuscript. YM and LH revised the manuscript. All authors contributed to the article and approved the submitted version.

## Author Disclaimer

The content of this article is solely the responsibility of the authors and does not necessarily represent the position of any funding body or initiative.

## Conflict of Interest

The authors declare that the research was conducted in the absence of any commercial or financial relationships that could be construed as a potential conflict of interest.

## Publisher’s Note

All claims expressed in this article are solely those of the authors and do not necessarily represent those of their affiliated organizations, or those of the publisher, the editors and the reviewers. Any product that may be evaluated in this article, or claim that may be made by its manufacturer, is not guaranteed or endorsed by the publisher.
